# *Schistosoma mansoni* Fibroblast Growth Factor Receptor A Orchestrates Multiple Functions in Schistosome Biology and in the Host-Parasite Interplay

**DOI:** 10.3389/fimmu.2022.868077

**Published:** 2022-06-22

**Authors:** Xiaofeng Du, Donald P. McManus, Conor E. Fogarty, Malcolm K. Jones, Hong You

**Affiliations:** ^1^ Infection and Inflammation Program, QIMR Berghofer Medical Research Institute, Brisbane, QLD, Australia; ^2^ Faculty of Medicine, The University of Queensland, Brisbane, QLD, Australia; ^3^ Genecology Research Centre, University of the Sunshine Coast, Brisbane, QLD, Australia; ^4^ School of Veterinary Science, The University of Queensland, Gatton, QLD, Australia

**Keywords:** *Schistosoma mansoni*, fibroblast growth factor receptor A, stem cell marker, immunolocalization, host-parasite interplay

## Abstract

Stem cells play significant roles in driving the complex life cycle of *Schistosoma mansoni*. Fibroblast growth factor (FGF) receptor A (SmFGFRA) is essential for maintaining the integrity of schistosome stem cells. Using immunolocalization, we demonstrated that SmFGFRA was distributed abundantly in germinal/stem cells of different *S. mansoni* life stages including eggs, miracidia, cercariae, schistosomula and adult worms. Indeed, SmFGFRA was also localized amply in embryonic cells and in the perinuclear region of immature eggs; von Lichtenberg’s layer and the neural mass of mature eggs; the ciliated surface and neural mass of miracidia; the tegument cytosol of cercariae, schistosomula and adult worms; and was present in abundance in the testis and vitellaria of adult worms of *S. mansoni*. The distribution pattern of SmFGFRA illustrates the importance of this molecule in maintaining stem cells, development of the nervous and reproductive system of schistosomes, and in the host-parasite interplay. We showed SmFGFRA can bind human FGFs, activating the mitogen activated protein kinase (MAPK) pathway of adult worms *in vitro*. Inhibition of FGF signaling by the specific tyrosine kinase inhibitor BIBF 1120 significantly reduced egg hatching ability and affected the behavior of miracidia hatched from the treated eggs, emphasizing the importance of FGF signaling in driving the life cycle of *S. mansoni*. Our findings provide increased understanding of the complex schistosome life cycle and host-parasite interactions, indicating components of the FGF signaling pathway may represent promising targets for developing new interventions against schistosomiasis.

## Introduction

Schistosomiasis is first on the scale of devastating parasitic helminth diseases, causing more than 250 million human infections in 74 countries and leading to considerable morbidity and an estimated loss of 1.9 million disability-adjusted life years (1–3). There are three main schistosome species (*Schistosoma mansoni, S. japonicum* and *S. haematobium*) causing human schistosomiasis. Currently no human vaccines are available and, as clinical treatment relies entirely on the single drug praziquantel, the potential emergence of drug resistance is an ever-present concern (1). Recent studies have demonstrated that stem cells play vital roles in driving and maintaining the complex schistosome life cycle (4–8). Adult schistosomes parasitize mammals and lay eggs many of which eventually escape these definitive hosts in feces or urine. An egg gives rise to a ciliated larva, the miracidium, which infects a specific freshwater snail host and then undergoes a dramatic body conversion to produce an obligate asexually-reproducing parasitic stage, the mother sporocyst. Endogenous proliferation of stem cells in mother sporocysts leads to a new asexual stage - the daughter sporocyst. These ‘daughters’, in turn, can generate by stem cell proliferation either more daughters, or the next stage, the migratory cercaria (-ae), that escapes from the snail into the aquatic environment. The cercariae then penetrate the skin of a mammalian host to transform into a schistosomulum (-a), before entering the host vascular system. These juvenile schistosomes develop a functional digestive system and sexual reproductive organs through *de novo* processes that commence by differentiation of pluripotent stem cells (6), early in the development of schistosomula. The larvae then mature into sexual dimorphic adults and the female worms lay eggs into the mesenteric venules of the host (9). Given the critical roles that stem cells play in schistosome biology (10), targeting genes vital for controlling the integrity of these cells may, by impeding parasite development at critical phases of the life cycle, provide a novel strategy for drug and/or vaccine development against schistosomiasis.

The fibroblast growth factor (FGF) signaling pathway in mammals is critically involved in a variety of processes during embryonic development and adult homeostasis through promoting cell proliferation, cell differentiation, cell survival, tissue repair/regeneration, drug resistance, and apoptosis (7, 11–15). Significantly, FGF signaling is crucial in stem cell control in various model systems including both vertebrates (5, 16–18) and invertebrates (14, 19–22). The mammalian FGF pathway is activated by binding FGF ligands to FGFRs, thereby phosphorylating mitogen-activated protein kinase (MAPK), phosphoinositide 3-kinase (PI3K)/AKT (protein kinase B, also called PKB), phospholipase C gamma (PLCγ), and signal transducers and activators of transcription (STAT) (23–25). The FGF signalling pathway has been shown present in various multicellular organisms ranging from vertebrates, such as humans and mice (13, 26–31), to invertebrates including *Drosophila* (32, 33) and the free-living nematode, *Caenorhabditis elegans* (34, 35). The FGF signaling pathway has also been demonstrated in members of the animal clade Lophotrochozoa (6, 7, 14, 20, 36, 37). For the Platyhelminthes, flatworms capable of exquisite regenerative or generative capacity, the FGF pathway has been described in free-living triclad planarians, with expression of FGF receptors found in stem cells of Dugesia japonica (19, 20) and Schmidtea mediterranea (21, 22)*. Similarly, three* genes encoding FGFRs (*emfr1*, *emfr2*, *emfr3*) have been identified in the cestode *Echinococcus* *multilocularis*, a parasite capable of invasive growth in host livers as it undergoes internal asexual generation of abundant infectious tapeworm scoleces for infection of a new host (14). Expression patterns of the *E.* *multilocularis* FGFs suggested that only *emfr2* and *emfr3* could be expressed in the parasite’s stem cells or their immediate progeny, while *emfr1* did not have a typical stem cell-specific expression pattern (14). Whereas no endogenous FGF ligands were identified in *E*. *multilocularis*, human FGF ligands were shown able to activate all three *Echinococcus* FGFRs and stimulate parasite development *in vitro* (14), thereby highlighting the importance of the interaction between host-derived hormones and the corresponding receptors of evolutionarily conserved parasite signaling pathways (14).

To date, two FGFR-encoding genes (*SmfgfrA* and *SmfgfrB*) have been described in *S. mansoni* (6, 36). *SmfgfrA* is expressed in the germinal cells of larvae (4, 6) and the stem cells of adult *S. mansoni* (7, 38, 39), emphasizing the vital roles SmFGFRA plays in the maintenance and proliferation of *S. mansoni* stem cells (6, 7, 27, 40). Hahnel *et al.* found the transcriptional levels of both SmFGRFs were upregulated following adult worm pairing, indicative of their importance in schistosome reproduction (36).

The role played by FGF signaling in different schistosome life cycle stages is unclear. To address this issue, we examined the expression of SmFGFRA in different developmental stages of *S. mansoni* by real-time PCR and immunolocalization and explored the co-localization of SmFGFRA and stem cells in adult worms. Furthermore, by employing protein binding and phosphorylation assays, we determined the binding affinity between SmFGFRA and host FGF ligands and showed this binding can activate the MAPK pathway in *S. mansoni*. In addition, we assessed the effects of the inhibitor BIBF 1120 on the development of eggs and the behavior of hatched miracidia of *S. mansoni.* BIBF 1120 is a highly selective adenosine triphosphate (ATP)-competitive tyrosine kinase (TK) inhibitor that has been shown to significantly reduce the numbers of somatic stem cells in adult worms of *S. mansoni* (36).

## Materials and Methods

### Ethics

All experiments were approved by the Animal Ethics Committee (ethics number P242) of the QIMR Berghofer Medical Research Institute. The study was conducted according to the guidelines of the National Health and Medical Research Council of Australia, as published in the Australian Code of Practice for the Care and Use of Animals for Scientific Purposes, 7th edition, 2004 (www.nhmrc.gov.au). All work involving live *S. mansoni* life cycle stages was performed in quarantine-accredited laboratories as required by Australian Biosecurity law.

### Parasites

Swiss mice (female, 6 weeks old) were infected subcutaneously with 100 *S. mansoni* cercariae. Seven weeks post-infection the mice were euthanised and adult worms were obtained by portal perfusion with 37°C pre-warmed RPMI Medium 1640 (Gibco, Sydney, Australia). Mouse livers were removed at necropsy and liver eggs were isolated (41, 42), with immature and mature liver eggs separated as described (43). Miracidia were harvested by hatching mature eggs in deionized water under light (41). *S. mansoni* cercariae were obtained by shedding infected *Biomphalaria glabrata* snails under bright light. Schistosomula were obtained by mechanical transformation of cercariae *in vitro* as described (2), and cultured in Basch’s medium (2) for five days to produce 5-day old schistosomula.

### Sequence Analysis of SmFGFRs

Searches for SmFGFRs sequences were conducted using genome version Smansoni_v7, GCA_000237925.3 (44, 45) on the WormBase ParaSite website (https://parasite.wormbase.org/Schistosoma_mansoni_prjea36577/Info/Index). Searches for homologous FGFR protein sequences were performed using the Basic Local Alignment Search Tool (BLAST) on the NCBI website (http://blast.ncbi.nlm.nih.gov/Blast.cgi) and the WormBase ParaSite website (http://parasite.wormbase.org/Multi/Tools/Blast). Protein sequence identity was analyzed using EMBOSS Needle (https://www.ebi.ac.uk/Tools/psa/emboss_needle/) (46, 47). Signal peptides were predicted by the SignalP-5.0 Server (http://www.cbs.dtu.dk/services/SignalP/). Protein molecular weight and isoelectric point were calculated using the ExPASy-Compute pI/Mw tool (http://web.expasy.org/compute_pi/). Domain predictions were conducted using the Simple Modular Architecture Research Tool (SMART) (http://smart.embl-heidelberg.de/). Prediction of SmFGFRA 3D structures was performed using I-TASSER (https://zhanglab.dcmb.med.umich.edu/I-TASSER/) (48–50). Searching the *S. mansoni* single-cell atlas was carried out using the SchistoCyte resource (http://www.collinslab.org/schistocyte/) (39, 51).

### *SmfgfrA* Transcription Levels in Different Life Cycle Stages Determined by Real-Time PCR

Total RNAs were extracted from *S. mansoni* eggs, miracidia, cercariae, schistosomula and adult male and female worms using RNeasy Mini Kits (Qiagen, Melbourne, Australia), followed by cDNA synthesis using QuantiTect Reverse Transcription Kits (Qiagen). Quantitative real-time PCR (qPCR) was performed with QuantiNova SYBR^®^ Green PCR Kits (Qiagen) using a Corbett Rotor Gene 6000 Real-Time PCR system. Forward primer (5’-ATGGGACTCAATTACGCATT-3’) and reverse primer (5’-CACCACTGTCTTCCGACCTG-3’) for *SmfgfrA* were designed using the Primer 3 software (http://frodo.wi.mit.edu/), and the specificity of the primer sequences was confirmed by BLAST. The *S. mansoni* Glyceraldehyde-3-Phosphate Dehydrogenase (GAPDH) housekeeping gene was used as reference gene (52). The qPCR reactions comprised 10 µl 2xSYBR Green PCR Master Mix, 100 ng cDNA, and 0.7 µM of each primer. The cycling parameters were as follows: 95°C for 2 min, 40 cycles of 95°C for 5 s and 58°C for 10 s. Relative gene transcriptional levels were normalized to the GAPDH gene and determined using the 2^-ΔΔCt^ calculation.

### Protein Expression, Purification and Antibody Generation

The SmFGFRA extracellular ligand binding domain (from T^39^ to L^386^, SmFGFRA-L) coding sequence (excluding signal peptide) was amplified from *S. mansoni* cDNA using a forward primer (5’-TACTTCCAATCCAATGCAACTTTACACTGTGCGTGTGACGC-3’) and a reverse primer (5’-TTATCCACTTCCAATGTTATTATCACAATCCACTATCCCTATAGGACAAATTTTC-3’). The full length SmFGFRA (from L^27^ to H^918^) coding gene, without signal peptide, was amplified from *S. mansoni* cDNA using a forward primer (5’-TACTTCCAATCCAATGCACTTGAGTGTAAATCACAATCAATGTACGAA-3’) and a reverse primer (5’-TTATCCACTTCCAATGTTATTATCACTAGTGTAAATACTGTCGCGGTTCCAAGT-3’). Both fragments were expressed in *Escherichia coli* after cloning into the pET His6 TEV LIC cloning vector (1B) (a gift from Scott Gradia, Addgene, Watertown, Massachusetts, USA); a Ligation Independent Cloning (LIC) fusion tag was added at the 5’ end of each primer (underlined in each primer sequence). The vector was linearized using the SspI-HF restriction enzyme (New England Biolabs, Melbourne, Australia) and then purified using a QIAquick Gel Extraction Kit (Qiagen). The linearized vector and insert were treated with T4 polymerase and then annealed at a molecular ratio of 1:3 at room temperature for 5 min followed by the addition of 1 μl 25 mM EDTA to stop the reaction. After sequence confirmation, the reconstructed vectors were transformed into *E. coli* (Rosseta strain) for expression induced with 1 mM IPTG (isopropyl-β-D-thiogalactopyranoside) (Sigma-Aldrich, Sydney, Australia) at 37°C for 3 h. Recombinant protein was purified using a Ni-NTA His-tag affinity kit (Qiagen). HALT protease inhibitor cocktail (Thermo Fisher Scientific) was added during protein purification. The His-tag on the purified protein was then removed by ProTEV Plus (Promega, Sydney, Australia) according to the manufacturer’s instructions.

A polyclonal antibody against the recombinant SmFGFRA-L (rSmFGFRA-L) was generated in SWISS mice (8 weeks old, female). Briefly, five mice were injected intraperitoneally with rSmFGFRA-L protein (25 μg/each mouse) adjuvanted with Montanide ISA 720 VG (SEPPIC, Courbevoie, France) three times at 2-weekly intervals (3). Blood was collected 2 weeks after the third injection. The titre of the antibody was determined using an enzyme-linked immunosorbent assay (ELISA) as described (53–55). In brief, an ELISA plate (Thermo Fisher Scientific, Brisbane, Australia) was coated with rSmFGFRA-L protein (1 µg/ml, 100 µl/well) in 0.05 M Carbonate-Bicarbonate coating buffer (pH 9.6) overnight at 4°C, followed by blocking at 37°C for 1 h with blocking buffer [1% (w/v) bovine serum albumin (BSA) (Sigma-Aldrich) in PBS containing 0.05% (v/v) Tween-20 (PBST)]. Serially diluted serum in blocking buffer (100 µl/well) was added and the plate incubated at 37°C for 1 h. As negative controls, naïve mouse serum was used. Following 3 x washes with 0.05% PBST, a secondary antibody, Novex™ Goat anti-Mouse IgG (H+L) - cross-adsorbed, horseradish peroxidase (HRP) conjugate (Thermo Fisher Scientific), was added (1:2,000, 100 µl/well) and the plate was incubated at 37°C for 1 h. After 3 x washes with 0.05% PBST, 1-Step™ Ultra TMB-ELISA Substrate Solution (Thermo Fisher Scientific) was added (50 μl/well) followed by 10 min incubation at room temperature prior to stopping the reaction with 2 M sulphuric acid (50 μl/well). The absorbance of each well was measured at 450 nm using a POLARstar OPTIMA multi-detection microplate reader (BMG LABTECH, Victoria, Australia). A positive antibody response was defined as an OD450 higher than 2.1 times the mean of the OD450 of serum samples from control mice.

### Western Blot Analysis

*S. mansoni*, *S. japonicum*, and *S. haematobium* soluble worm antigen preparation (SWAP) (53) and *S. mansoni* soluble egg antigen (SEA) (56) were prepared as described. The mouse anti-rSmFGFRA-L serum was utilized in western blots to probe electrophoresed schistosome SWAP and *S. mansoni* SEA. Protein samples were separated on 12% SDS-PAGE gels and transferred to an Immun-Blot low fluorescence-PVDF membrane (Bio-rad, Sydney, Australia). The membrane was first blocked with Odyssey Blocking Buffer (PBS) (LI-COR Biosciences, Lincoln, Nebraska, USA) for 1 h at room temperature. Then, the membrane was incubated with the mouse anti-rSmFGFRA-L serum (1:100 diluted in Odyssey buffer with 0.1% Tween-20) for 1 h followed by four washes in phosphate-buffered saline (PBS) plus 0.1% Tween-20 (0.1% PBST). Subsequently, the membrane was incubated with IRDye-labeled 680LT goat anti-mouse IgG antibody (Li-COR Biosciences) (1:15,000 diluted in Odyssey buffer with 0.1% Tween-20 and 0.01% SDS) for 1 h with shaking in a dark chamber. After washing (4X) with 0.1% PBST, the membrane was dried in the dark and visualized using the Odyssey CLx Infrared Imaging System (53).

### Immunolocalization of SmFGFRA in Different Life Cycle Stages of *S. mansoni* and Co-Localization of SmFGFRA and Stem Cells

*S. mansoni* adult worms, immature eggs, mature eggs, miracidia, cercariae, and 5-day old schistosomula were fixed in 10% (w/v) formalin in PBS prior to paraffin embedding. Sections (3-4 µm) prepared from the paraffin blocks were affixed to positively charged adhesive slides, air-dried overnight at 37°C and then dewaxed and rehydrated through xylol and descending graded alcohols to water. Subsequently, the sections were transferred to Dako Target Retrieval Buffer (pH 9.0) (Dako, Carpinteria, California, USA) and subjected to 30 min heat antigen retrieval at 95°C. This was followed by washing three times in 0.1% PBST. Sections were blocked in Biocare Medical Background Sniper with 2% (w/v) BSA (Sigma-Aldrich) for 15 min to stop non-specific binding and then incubated at room temperature overnight with mouse anti-rSmFGFRA-L antibody (1:50 dilution). After washing three times with 0.1% PBST, the sections were incubated with Alexa Fluor^®^ 555 donkey anti-mouse IgG (Invitrogen, Melbourne, Australia) (1:300 dilution) for 2 h at room temperature.

To determine the co-localization of SmFGFRA and stem cells in adult *S. mansoni*, freshly perfused worms were cultured overnight in RPMI complete medium [RPMI Medium 1640 (Gibco, Sydney, Australia) supplemented with 10% (v/v) heat-inactivated fetal bovine serum (FBS, Gibco) and 100 IU/ml penicillin and 100 μg/ml streptomycin (Gibco)] at 37°C in 5% CO_2._ Then the worms were incubated for 24 h with 10 µM EdU (thymidine analog 5-ethynyl-2’-deoxyuridine) (Thermo Fisher Scientific), which only stains stem cells in schistosomes (7). The stained worms were fixed in 10% formalin, paraffin embedded and sectioned. Sections (4 µm) of EdU-labeled adult worms were subjected to EdU detection using a Click-iT™ EdU Cell Proliferation Kit (Alexa Fluor™ 488 dye) (Thermo Fisher Scientific) according to the manufacturers’ instructions. Then the same sections were subjected to SmFGFRA immunolocalization as described above. Nuclei in all tissue sections were also stained with diamidino-2-phenylindole (DAPI) gold (Invitrogen) and visualized using a Zeiss 780 NLO confocal microscope (Zeiss, Oberkochen, Germany).

### Protein Binding Assay

The binding affinity between rSmFGFRA-L/human FGFR1 and human acidic FGF (aFGF)/basic FGF (bFGF) was determined using the Octet RED 96 System (FortéBio, Menlo Park, California, USA) in standard Greiner black 96-well microplates (Sigma-Aldrich) as described (53). Briefly, to investigate the binding between rSmFGFRA-L and human aFGF/bFGF, the rSmFGFRA-L protein was biotinylated using a NHS-PEO4-biotin kit (Thermo Fisher Scientific), desalted with a Zeba Spin Desalting Column (Thermo Fisher Scientific), and then the protein was immobilized to a Streptavidin Biosensor (FortéBio). Prior to the assay, the Biosensors were hydrated in kinetic buffer (PBS with 15 mM NaCl, 0.1 mg/ml BSA, 0.002% Tween-20) for 60 min. Subsequently, a duplicate set of Biosensors were first incubated in kinetic buffer for 300 s as baseline and followed by immobilization for 600 s in 200 μl of 150 ng/μl biotinylated rSmFGFRA-L protein. Next, the Biosensors were washed in kinetic buffer for another 300 s. Finally, the sensors were exposed to a series (200 μl volume) of diluted concentrations of human aFGF/bFGF (Thermo Fisher Scientific). To further explore whether the binding affinity between human bFGF and rSmFGFRA-L is comparable to that between human bFGF and human FGFR1, human bFGF (8 ng/μl) was biotinylated and immobilized to Biosensors and then exposed to different concentrations (30 ng/μl and 23 ng/μl) of rSmFGFR-L and recombinant extracellular ligand binding domain of human FGFR1 (R^22^-I^376^) (FGFR1-L) (Sigma-Aldrich). PBS was used as a negative control. The association of the two proteins was detected for 1000 s followed by dissociation in the kinetic buffer for 1000 s. Experiments were run at 30°C with the orbital agitation of the microplate set to 1000 rpm. Data were analyzed using the FortéBio Data Analysis 7.1 program and included a double reference subtraction. The sample subtraction was conducted using PBS as a reference control, and sensor subtraction was performed on all samples automatically (53).

### Detection of MAPK Using an Anti-Phospho-P44/42 MAPK (Erk1/2) Antibody

An anti-phospho-P44/42 MAPK (Erk1/2) (Thr202/Tyr204) antibody (Cell Signaling Technology, New England Biolabs, Ipswich, USA) was used to detect activated (phosphorylated) extracellular signal regulated kinases 1 and 2 (Erk1/2) in *S. mansoni* adult worms (53) following stimulation with human aFGF or bFGF. Briefly, freshly perfused *S. mansoni* adult worms were cultured overnight in RPMI complete medium at 37°C in 5% CO_2._ The parasites were then cultivated for 30 min in RPMI complete medium containing 10 nM human aFGF, or 10 nM bFGF, or 10 nM aFGF and bFGF (the same concentration used in a previous study (14)). For the inhibitor treatment group, adult *S. mansoni* were cultured in RPMI complete medium with 10 μM BIBF 1120 (dissolved in dimethyl sulfoxide (DMSO) and then diluted to different concentrations) (Sigma-Aldrich), or 0.1% DMSO (negative control) for 30 min as described (14). After each treatment, worms were harvested for SWAP extraction as described (53) in the presence of HALT protease inhibitor cocktail. Isolated SWAP was then separated on a 10% SDS-PAGE gel and analyzed by western blotting as described above.

### Tyrosine Kinase Activity Assay

A Universal Tyrosine Kinase Assay Kit (Takara, Melbourne, Australia) was used to determine the enzymatic activity of rSmFGFRA at different concentrations (100 ng/μl - 0.78 ng/μl) in the presence or absence of 5 μM and 10 μM BIBF 1120 as described (14, 36), following the manufacturer’s instructions. DMSO (0.1%) was served as negative control. The activity of rSmFGFRA was determined by comparing its absorbance with that of control protein tyrosine kinase (PTK), supplied with the kit, according to the manufacturer’s instructions. Technical duplicates were performed and the experiment was repeated twice.

### BIBF 1120 Treatment of *S. mansoni* Eggs *In Vitro* and Measurement of the Behavior of Miracidia Hatched From the Treated Eggs

*S. mansoni* eggs were cultured overnight at 37°C in RPMI complete medium under an atmosphere of 5% CO_2._ The eggs were then treated with different concentrations of BIBF 1120 (2.5 μM, 5 μM, 10 μM, 20 μM) or 0.1% DMSO (negative control). After seven days, the eggs were collected and miracidia were hatched in deionized water under light as described above. The egg hatching efficiency (%) in each group was calculated by dividing the number of hatched eggs with the total number of examined eggs X 100. The behavior of miracidia hatched from treated and control eggs was also monitored using a published bioassay (57, 58). Briefly, around 30 *S. mansoni* miracidia in 100 μl deionized water were, using a pipette, evenly distributed to the centre of a microscope slide. Miracidial movement (swimming) was monitored using an Olympus-CKX41 microscope equipped with an Olympus DPI Digital Microscope Camera DP22 (25 frames per second at 2.8-megapixel image quality). Miracidial movement in the field of view (FOV) was recorded for 1 minute by video. Then the videoed miracidial tracks were analyzed using FIJI software to calculate three individual behavioral measurements including velocity of miracidial movement, duration (time) of miracidia staying within the FOV, and tortuosity (the ratio of track length to maximum displacement) of miracidial movement (57). The miracidial movement velocity was calculated in pixel/s using the rolling mean subtraction method (57, 58). Miracidial location was tracked in each frame along an x-y axis and the trajectories were interpolated using the plugin for FIJI software, known as TrackMate (58, 59). Employing the MTrackJ plugin, the average velocity, duration, and tortuosity of miracidia in the FOV were determined. Heatmaps, representing the movement pattern of individual miracidia, were generated as described (58, 60).

### Statistical Analysis

All data are displayed as the mean ± SE. Differences between groups were analyzed for statistical significance by One-way ANOVA. GraphPad Prism software (Version 8.2.1, La Jolla, CA, USA) was used for all statistical analyses. A statistically significant difference for a particular comparison was defined as a *p* value ≤ 0.05. * *p* value≤ 0.05, ** *p* value≤ 0.01, *** *p* value ≤ 0.001, **** *p* value ≤ 0.0001, not significant (ns).

## Results

### SmFGFRA Sequence and Structural Analysis

*SmfgfrA* (Smp_175590) encodes SmFGFRA and *SmfgfrB* (Smp_157300) has two transcripts encoding SmFGFRB1 and SmFGFRB2, respectively. SmFGFRA shares 78.4% amino acid sequence identity with *S. haematobium* FGFR2 (ShFGFR2, MS3_0015372) and 59.9% identity with *S. japonicum* FGFR2 isoform 2 (SjFGFR2-2, EWB00_002899). In contrast, SmFGFRA shares only 17.4% and 17.9% amino acid sequence identity with SmFGFRB1 and SmFGFRB2, respectively, and their common amino acids are mainly distributed in the conserved TK domain. SmFGFRA shares 21.7% sequence identity with human FGFR1.

**Figure 1:**
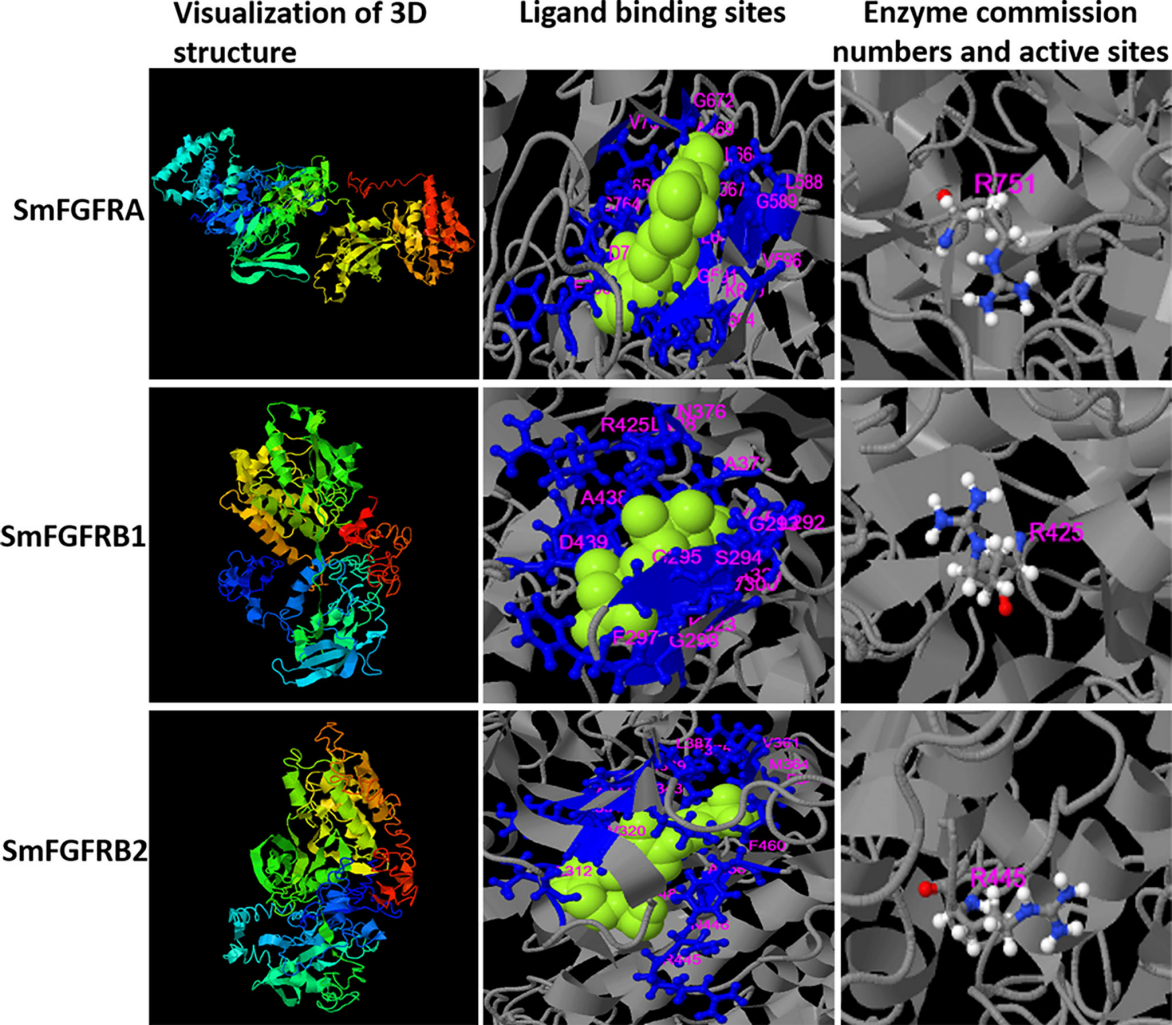
Three-dimensional (3D) structure (column 1), the predicted binding ligands (green spheres) and binding residues (blue balls and sticks) (column 2), and enzyme active sites (colored balls and sticks) (column 3) for *S. mansoni* FGFRA (Confidence score=-2.22), FGFRB1 (Confidence score=-2.7) and FGFRB2 (Confidence score=-3.74) predicted by the I-TASSER server. The residue numbers are shown in pink.

The tertiary structures of the SmFGFRs were predicted by the I-TASSER server (**Figure 1**). The ligand binding residues (L^588^, G^589^, G^591^, V^596^, A^617^, K^619^, I^650^, R^651^, F^652^, I^664^, L^666^ - A^669^, G^672^, V^754^, C^764^, D^765^, F^766^) and the enzyme active site residue (R^751^) of SmFGFRA were predicted as shown in **Figure 1**. The ligand binding residues (l^292^ - A^295^, G^297^, I^298^, Y^300^, A^321^, K^323^, I^353^, M^369^, E^370^ -A^372^, N^376^, R^425^, L^428^, A^438^, D^439^) and enzyme active site residue (R^425^) of SmFGFRB1, and the ligand binding residues (L^312^, V^320^, A^341^, K^343^, E^360^, V^361^, M^364^, I^373^, F^375^, L^387^, M^389^, E^390^-A^392^, R^445^, N^446^, L^448^, A^458^, F^460^) and the enzyme active site residue (R^445^) of SmFGFRB2 are shown in **Figure 1** (14). The predicted domain structures of SmFGFRA and SmFGFRB1/2 are shown in Supplementary **Figure 1**. Neither signal peptides nor immunoglobulin (IG)-like domains that are responsible for protein-protein and protein-ligand interactions (61, 62) were found in either SmFGFRB1 or SmFGFRB2 (**Supplementary Figure 1**).

The predicted binding ability of SmFGFRA with its ligand indicated its potential in inducing dimerization of the extracellular domains of FGFRs [as demonstrated in mammalian cells (11, 12, 63)], and subsequent transautophosphorylation of the cytoplasmic TK domain, thereby activating downstream signal transduction in *S. mansoni*. However, no endogenous genes encoding FGF ligands have been identified in the genomes of *S. mansoni* (45, 64), suggesting that the parasite might explore host FGF ligands to stimulate its own signaling pathway.

### Quantification of *SmfgfrA* Transcripts in Different *S. mansoni* Life Stages

**Figure 2:**
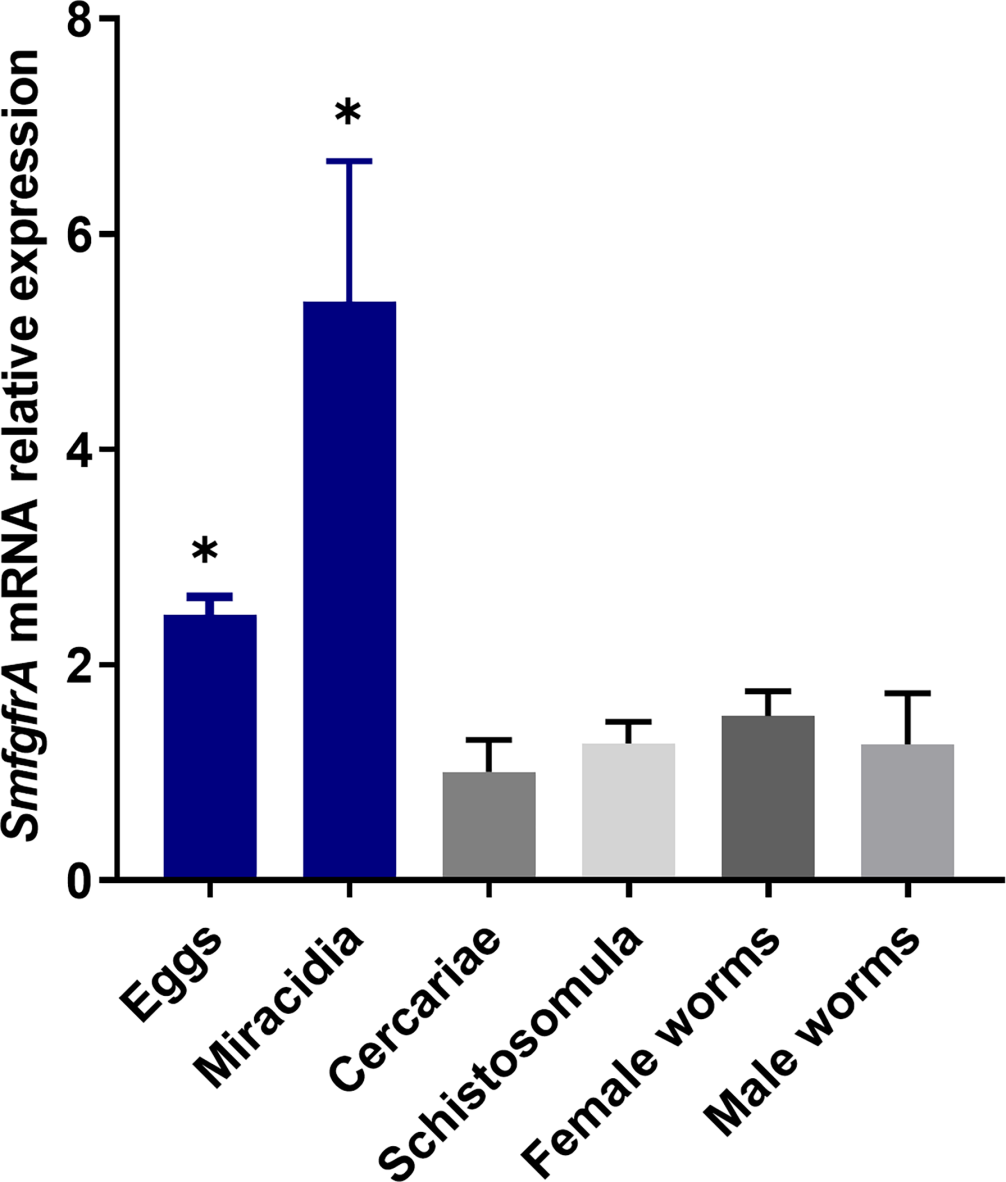
Quantification of *SmfgfrA* gene transcripts in different life cycle stages of *S. mansoni.* The relative expression of *SmfgfrA* in eggs, miracidia, cercariae, schistosomula, and adult female and male worms were normalized to its expression in cercariae. Data are representative of the mean ± SE of three independent experiments. *P* values were calculated using cercariae as a comparison group. (*p value≤ 0.05, One-way ANOVA).

Using real-time PCR assays, transcriptional levels of the *SmfgfrA* gene were quantified in eggs, miracidia, cercariae, schistosomula, and adult female and male worms of *S. mansoni*. We found *SmfgfrA* was transcribed in all the life cycle stages examined with eggs and miracidia having the highest expression levels (**Figure 2**).

### Purification of Recombinant SmFGFRA and Antibody Generation

**Figure 3:**
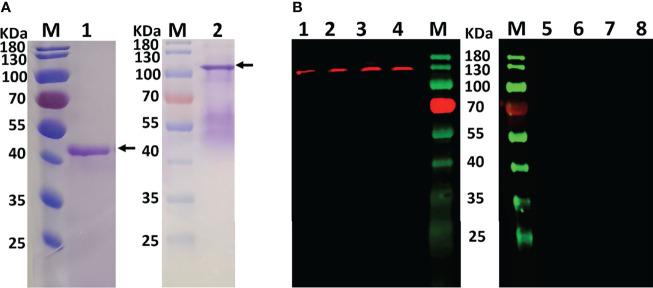
**(A)** Purified rSmFGFRA-L (lane1) and rSmFGFRA (lane 2) observed in SDS-PAGE gels. **(B)** Western blot analysis using an anti-rSmFGFRA-L polyclonal antibody to probe *S. mansoni* SWAP (lane1), *S. japonicum* SWAP (lane 2), *S. haematobium* SWAP (lane 3), and *S. mansoni* SEA (lane 4). The naïve mouse serum served as a negative control to probe *S. mansoni* SWAP (lane 5), *S. japonicum* SWAP (lane 6), *S. haematobium* SWAP (lane 7), and *S. mansoni* SEA (lane 8).

Purified rSmFGFRA-L and rSmFGFRA, expressed in *E. coli*, were shown by SDS-PAGE to migrate as bands at the predicted sizes of approximately 40 KDa (**Figure 3A**, Lane 1) and 105 KDa (**Figure 3A**, Lane 2), respectively. The smeared bands observed in the SDS-PAGE with purified full-length protein rSmFGFRA (**Figure 3A**, Lane 2) may indicate protein degradation during protein expression and/or purification, although cocktail protease inhibitors were added during the purification procedure.

Specific antibody against rSmFGFRA-L was generated in mice and the titer of the antibody (1:25,600) determined by ELISA. The anti-rSmFGFRA-L serum was used for western blot analysis and immunolocalization.

### Reactivity of the Anti-rSmFGFRA-L Serum

To determine whether the anti-serum raised against SmFGFRA cross reacted with homologous components in SWAP extracts prepared from adult *S. japonicum* and *S. haematobium*, western blot analysis was performed using the generated mouse anti-rSmFGFRA-L serum. Western blots demonstrated that the anti-rSmFGFRA-L antibody (1:80) recognized a band at the expected molecular size of approximately 130 kDa in the SWAPs (50 μg/well) of *S. mansoni*, *S. japonicum* and *S. haematobium*, whereas no bands were evident when the SWAPs were probed with naïve mouse serum (**Figure 3B**). Similarly, native SmFGFRA (approximately 130 kDa) was also recognized by the anti-rSmFGFRA-L antibody in *S. mansoni* SEA while no band was recognized by the naïve mouse serum (**Figure 3B**).

### Localization of SmFGFRA in Different Life Cycle Stages of *S. mansoni* and Co-Localization of SmFGFRA and Stem Cells

**Figure 4:**
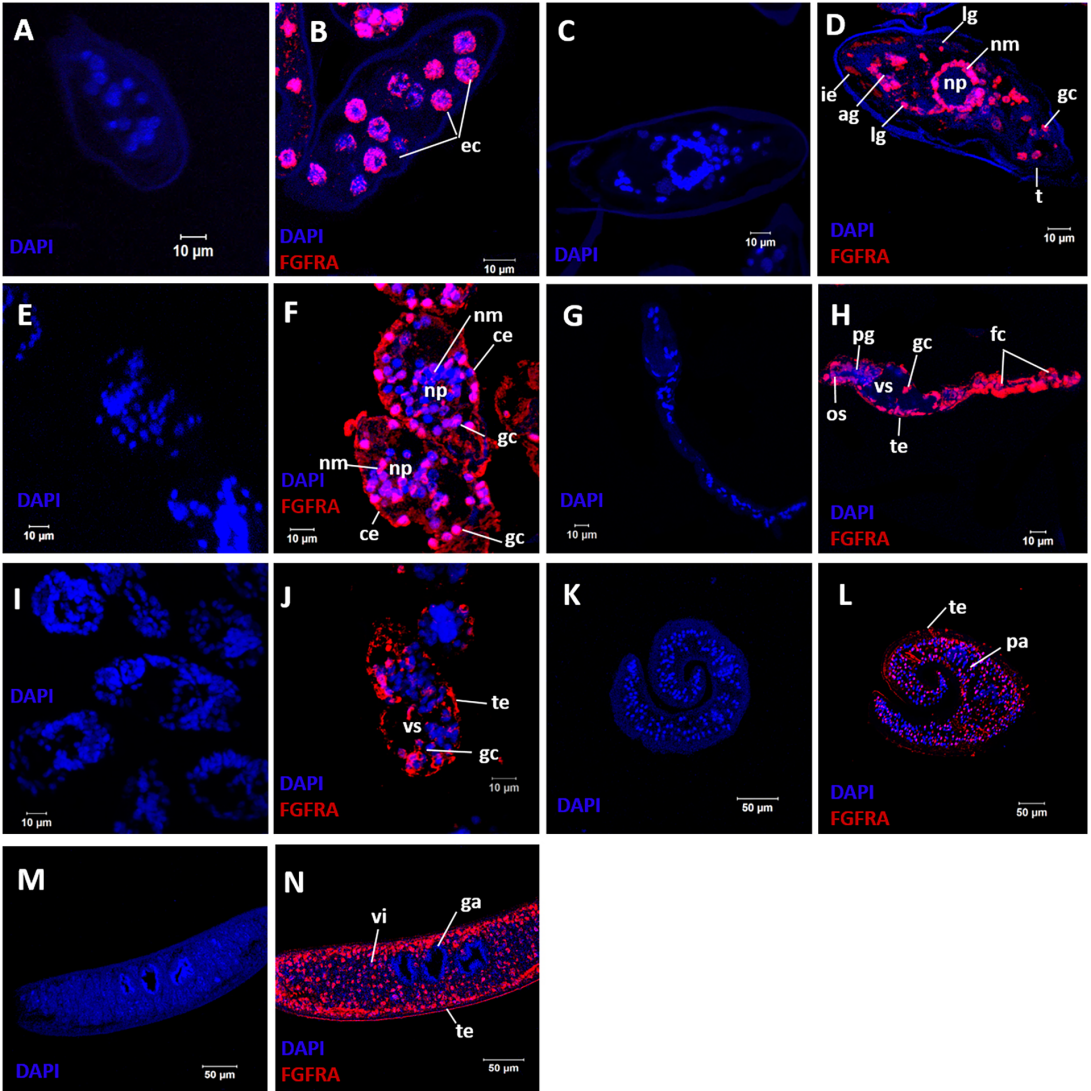
Immunolocalization of SmFGFRA in different life cycle stages of *S. mansoni*. Sections of different *S. mansoni* stages were exposed to a mouse anti-rSmFGFRA-L antibody to examine the distribution of SmFGFRA (shown in red). Naïve mouse serum was employed as negative control. All samples were DAPI stained (in blue). Fixed immature eggs were incubated with naïve control mouse serum **(A)** and mouse anti-rSmFGFRA-L antibody **(B)**; mature eggs were exposed to naïve control mouse serum **(C)** and mouse anti-rSmFGFRA-L antibody **(D)**; miracidia were probed with naïve control mouse serum **(E)** and mouse anti-rSmFGFRA-L antibody **(F)**; cercariae were incubated with naïve control mouse serum **(G)** and mouse anti-rSmFGFRA-L antibody **(H)**; schistosomula were exposed to naïve control mouse serum **(I)** and mouse anti-rSmFGFRA-L antibody **(J)**; adult male worms were probed with naïve control mouse serum **(K)** and mouse anti-rSmFGFRA-L antibody **(L)**; adult female worms were exposed to naïve control mouse serum **(M)** and mouse anti-rSmFGFRA-L antibody **(N)**. ec, embryonic cells; ie, inner envelop; ig, lateral glands; ag, apical gland; nm, neural mass primordium; np, neuropile; gc, germinal cells; ce, ciliated epithelium; os, oral sucker; pg, preacetabular glands; fc, flame cells; te, tegument; vs, ventral sucker; pa, parenchyma; vi, vitellarium; ga, gastrodermis.

**Figure 5:**
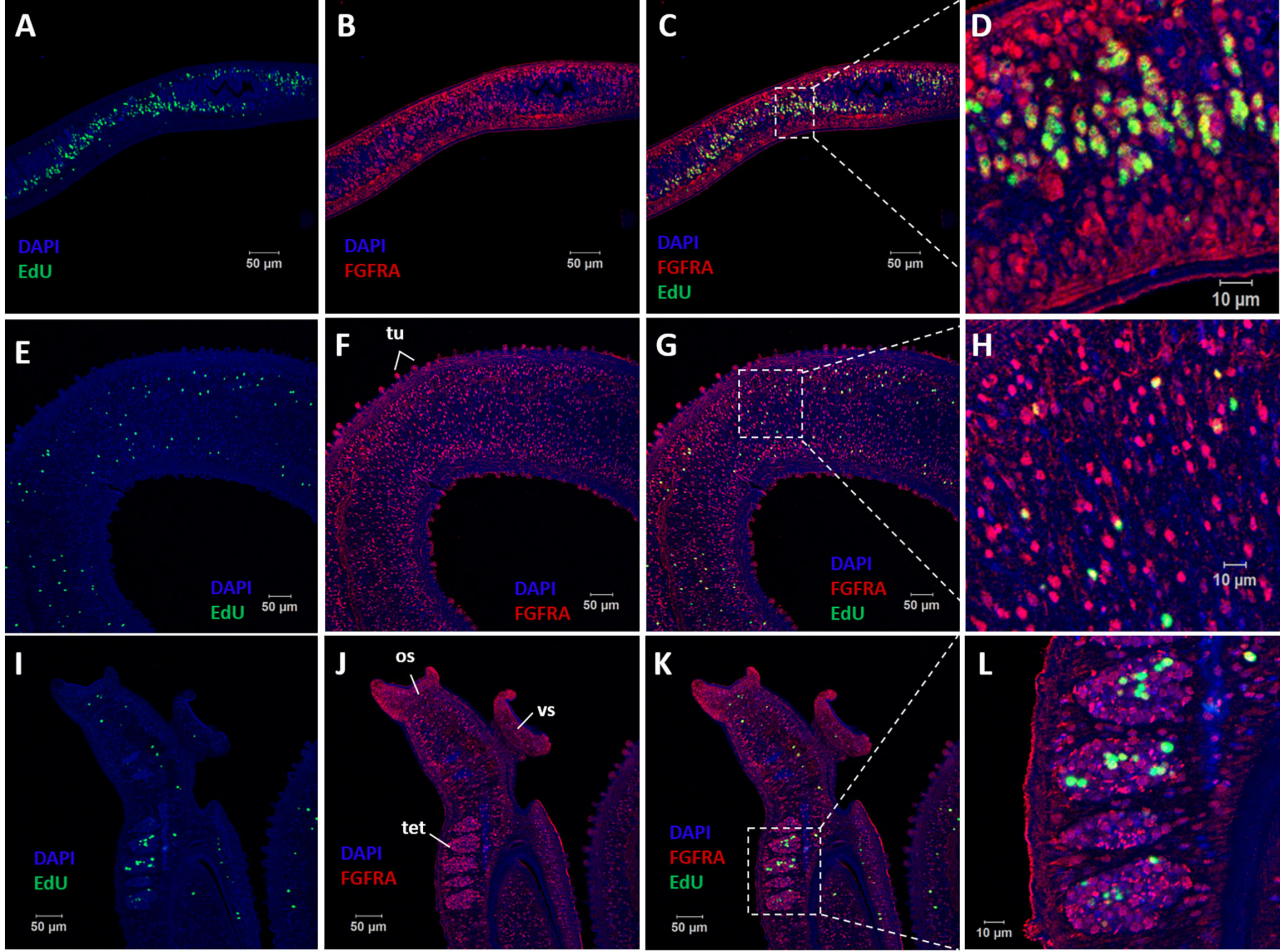
Co-localization of SmFGFRA and EdU^+^ cells in *S. mansoni* adult worms. Sequential staining of **(A)** EdU (green) and **(B)** anti-rSmFGFRA-L antibody (red) to sections of adult female worms. **(C)** Merging of **(A, B)**. **(D)** Magnified squared-areas in **(C)**. Sections of adult male worms were stained with **(E, I)** EdU and probed with **(F, J)** anti-rSmFGFRA-L antibody. **(G)** Merging of **(E, F)**. **(H)** Magnified squared-areas in **(G)**. **(K)** Merging of **(I, J)**. **(L)** Magnified squared-areas in **(K)**. All samples were DAPI-stained (blue). os, oral sucker; vs, ventral sucker; tet, testes; tu, tubercles.

Immunolocalization was performed on sections of immature and mature eggs collected from *S. mansoni* infected mouse livers, miracidia, cercariae, 5-day old schistosomula, and adult male and female worms to determine the distribution of SmFGFRA in the different developmental stages. Immunofluorescence showed that native SmFGFRA was localized in the embryonic cells (**Figure 4B**) of immature eggs (egg embryogenesis stages 2-3) (65, 66). In positive cells, the SmFGFRA signal was detected in a perinuclear distribution. In mature eggs, SmFGFRA was detected in the inner envelope (von Lichtenburg’s Layer), the peripheral cellular neural mass, the lateral and apical glands, and germinal cells (**Figure 4D**). SmFGFRA localization was not detected on the egg shell or in the central neuropile of the neural mass where no neuronal cells are present (67). The distribution of SmFGFRA was similar in miracidia to that observed in mature eggs being localized within the ciliated epithelium, neural mass, lateral glands, apical gland, and germinal cells (**Figure 4F**). SmFGFRA was also detectable in the tegument, oral sucker, preacetabular glands, germinal cells, and flame cells of cercariae (6, 68) (**Figure 4H**), and in the tegument and the internal cell masses, including germinal cells (4), of 5-day old schistosomula (**Figure 4J**). Significantly, SmFGFRA fluorescence labelling was detected in almost all tissues of the adult male worms including the tubercles (**Figure 5F**), tegument, parenchyma (**Figure 4L**), oral sucker, ventral sucker, and testes (69) (**Figure 5J**). Similarly, SmFGFRA was amply distributed in the tegument and inner cell masses of female worms including the vitellaria, but not the gastrodermis (**Figure 4N**). Naïve mouse serum served as negative control to probe fixed immature eggs (**Figure 4A**), mature eggs (**Figure 4C**), miracidia (**Figure 4E**), cercariae (**Figure 4G**), schistosomula (**Figure 4I**), adult male worms (**Figure 4K**) and female worms (**Figure 4M**).

EdU is known for its ability to incorporate into newly synthesized cellular DNA, and stains only proliferating stem cells in *S. mansoni* adults (7). To determine the relationship between EdU^+^ cells (stem cells) and cells that express SmFGFRA (SmFGFRA^+^), we undertook immunolocalization of SmFGFRA using an anti-rSmFGFRA-L polyclonal antibody to probe sections of adult worms stained with EdU. All EdU^+^ cells present in both adult female worms and male worms were SmFGFRA^+^ (**Figure 5**), emphasizing the critical roles of SmFGFRA in maintaining stem cells at the translational level. However, not all SmFGFRA^+^ cells were EdU^+^, suggesting SmFGFRA is multi-functional in *S. mansoni*, being not only important in stem cell maintenance but also being involved in other critical biological processes.

### SmFGFRA Has Strong Binding Affinity With Human bFGF

**Figure 6:**
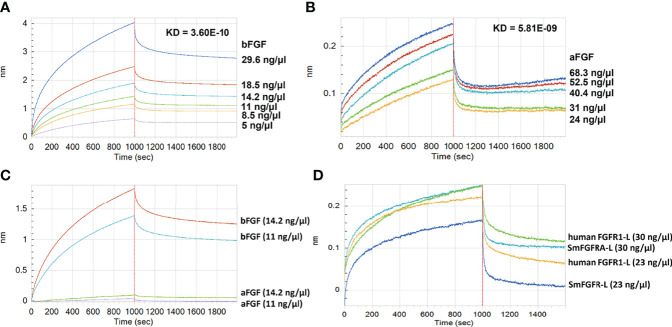
Assays showing Binding between rSmFGFRA-L and human bFGF/aFGF using the Octet RED system. The real-time binding response between rSmFGFRA-L and **(A)** human bFGF and **(B)** human aFGF at different concentrations (ng/μl) was monitored in seconds. The parameters of the binding affinity (nm) and the KD value (M) of the binding affinity between rSmFGFRA-L and human bFGF/aFGF are shown. **(C)** Comparison of the rSmFGFRA-L binding affinity to human bFGF and aFGF at concentrations of 14.2 ng/μl and 11 ng/μl, respectively. **(D)** Comparison of the human bFGF binding affinity to human FGFR1-L and rSmFGFRA-L at concentrations of 30 ng/μl and 24 ng/μl, respectively.

We performed real-time binding assays using the Octet RED system with ‘biolayer interferometry’ technology to investigate the binding affinity between rSmFGFRA-L and human bFGF/aFGF (**Figure 6**). A strong *in vitro* interaction between rSmFGFRA-L and human bFGF (protein concentration ranging from 5 ng/μl to 29.6 ng/μl) was detected (**Figure 6A**) (KD = 3.6E-10, coefficient of determination (r^2^) = 0.98). Binding affinity was also evident, albeit less strongly, between rSmFGFRA-L and human aFGF (protein concentration ranging from 24 ng/μl to 68.3 ng/μl) (KD = 5.81E-09, r^2^ = 0.92) (**Figure 6B**). Increasing the concentration of bFGF/aFGF resulted in an increased binding response with the dissociation phase slowly decreasing, demonstrating the specific binding ability presented between rSmFGFRA-L and bFGF/aFGF. To compare the binding affinity of rSmFGFRA-L to human bFGF and aFGF *in vitro*, rSmFGFRA-L immobilized sensors were exposed to bFGF or aFGF at 14.2 ng/μl and 11 ng/μl, respectively. At the same protein concentration, bFGF exhibited 19-33.7 times stronger binding affinity than aFGF to rSmFGFRA-L (**Figure 6C**). Notably, at the same concentration, human FGFR1-L demonstrated higher binding affinity than rSmFGFRA-L to human bFGF (**Figure 6D**).

### Human FGFs Activate the *S. mansoni* MAPK Signaling Pathway

**Figure 7:**
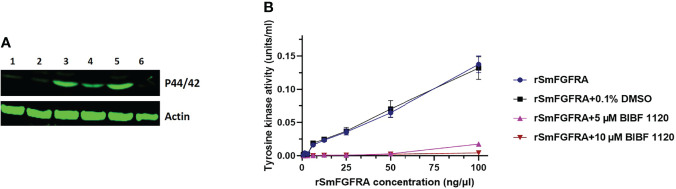
**(A)** Effect of human aFGF or bFGF on the stimulation of extracellular signal regulated kinases 1 and 2 (Erk1/2) in adult *S. mansoni* worms. An anti-phospho p44/42 MAPK (Erk) antibody (upper panel) and an anti-actin antibody (lower panel) were used to probe a protein extract of wild type, untreated adult worms (Lane 1) and worms which were incubated with 0.1% DMSO (Lane 2), human bFGF (Lane 3), human aFGF (Lane 4), both human bFGF and aFGF (Lane 5), and 10 μM BIBF 1120 (Lane 6), respectively. **(B)** Tyrosine kinase activity of rSmFGFRA at different concentrations (0-100 ng/µl) in the presence or absence of 0.1% DMSO or BIBF 1120 (5 µM and 10 µM).

To explore whether human FGFs could activate the *S. mansoni* MAPK pathway by phosphorylating Erk1/2, we incubated adult worms for 30 minutes with either 10 nM aFGF or bFGF individually or in combination. Then, SWAP extracted from the treated parasites was subjected to western blot analysis using an anti-phospho-P44/42 MAPK (Erk1/2) (Thr202/Tyr204) antibody. Phosphorylation of Erk1/2 was readily detected at the expected band size (approximately 44 kDa) (53) in SWAP of adult *S. mansoni* stimulated with either aFGF or bFGF, or co-stimulated with both aFGF and bFGF; stronger band intensity was observed in worms treated with bFGF (**Figures 7A**, **S2**). The Erk band was detectable in an extract of control parasites (wild type, i.e. untreated worms; and worms incubated with 0.1% DMSO). By using an anti-actin (control) antibody, no significant differences were evident in band intensity detected in any of the tested or control groups (**Figure 7A**, **S2**). Furthermore, 30 minute treatment of adult worms with the TK inhibitor BIBF 1120 (10 μM), an agent considered to selectively inhibit *S. mansoni* FGF receptors (36), resulted in markedly depleted phosphorylation of Erk (**Figure 7A**, **s2**).

### Tyrosine Kinase Activity of rSmFGFRA

SmFGFRA is a receptor tyrosine kinase (RTK) and the RTKs are important enzymes involved in the signal transduction pathway (12). A universal Tyrosine Assay Kit, used to measure the TK activity of rSmFGFRA, indicated, as might be expected, increased activity with increasing concentrations (0-100 ng/μl) of rSmFGFRA (**Figure 7B**). However, the TK activity of rSmFGFRA was considerably inhibited following incubation with the TK inhibitor, BIBF 1120; indeed, when the rSmFGFRA concentration was less than 6.25 ng/μl, TK activity was completely inhibited in the presence of 5 μM or 10 μM BIBF 1120 (**Figure 7B**).

### BIBF 1120 Inhibits the Hatching of *S. mansoni* Liver Eggs

**Figure 8:**
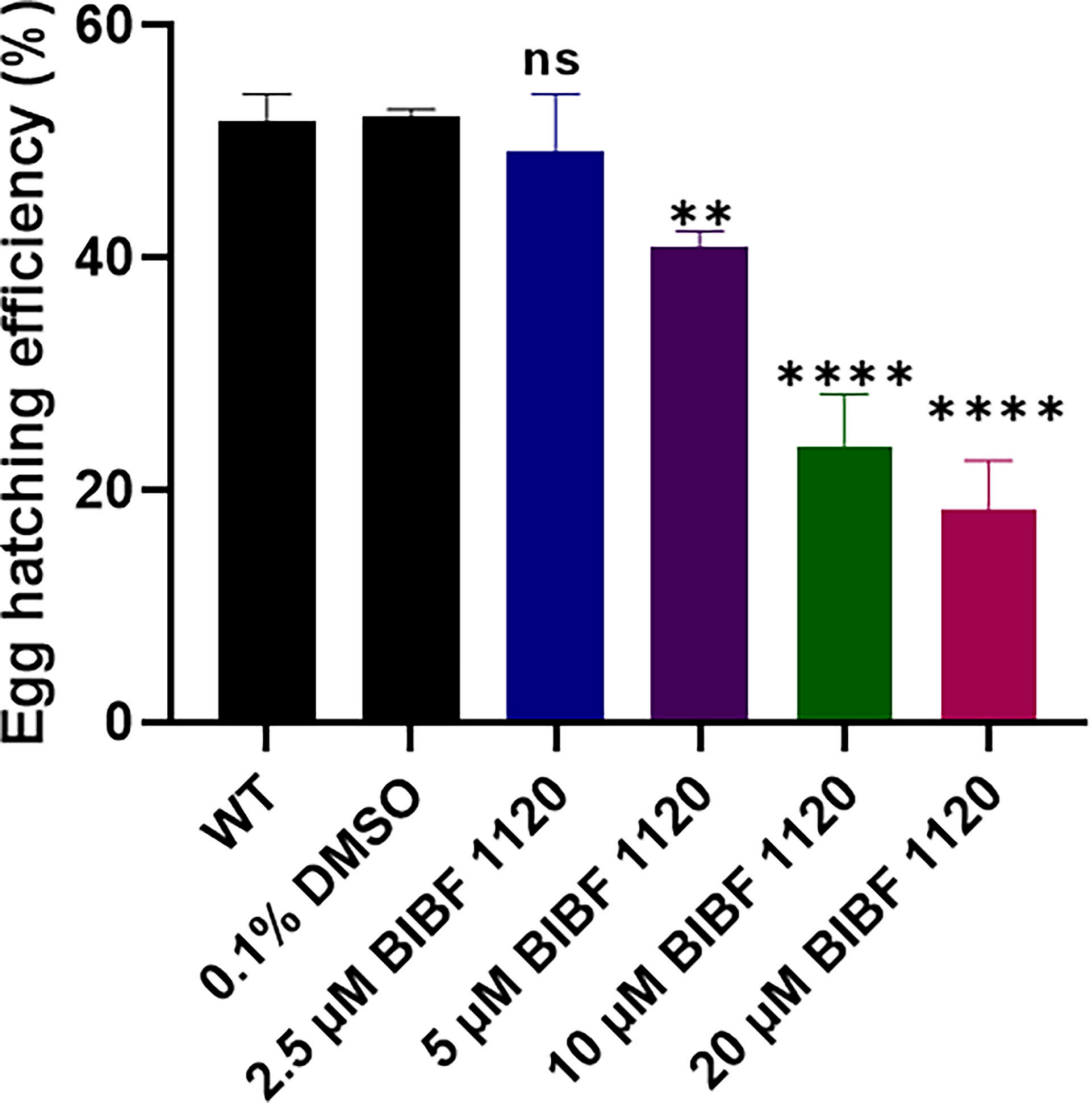
Effects of BIBF 1120 on the hatching of *S. mansoni* eggs. The egg hatching efficiency (%) of untreated wild type (WT) *S. mansoni* eggs and eggs treated with 0.1% DMSO, or 2.5 μM, 5 μM, 10 μM, and 20 μM BIBF 1120 was calculated by dividing the number of hatched eggs by the total number of eggs (hatched and unhatched eggs) X 100. (ns, not significant; **p value≤ 0.01, ****p value ≤ 0.0001 by One-way ANOVA).

To further understand the effects of inhibiting the FGFRs by BIBF 1120 in *S. mansoni*, eggs isolated from the livers of infected mice were treated *in vitro* with different concentrations (2.5 - 20 μM) of the inhibitor, and then the egg hatching efficiency was examined. BIBF 1120 had clear concentration-dependent effects on egg hatching ability. In the presence of 2.5 μM BIBF 1120, egg hatching efficiency was moderately but not significantly reduced. However, egg hatching was markedly decreased by 21.3% (*p*=0.0077), 54.4% (*p ≤* 0.0001), and 65% (*p ≤* 0.0001) in the presence of 5 μM, 10 μM, and 20 μM BIBF 1120, respectively, compared with control eggs treated with 0.1% DMSO (**Figure 8**).

### Behavioral Changes of *S. mansoni* Miracidia Hatched From Liver Eggs Treated With BIBF 1120

**Figure 9:**
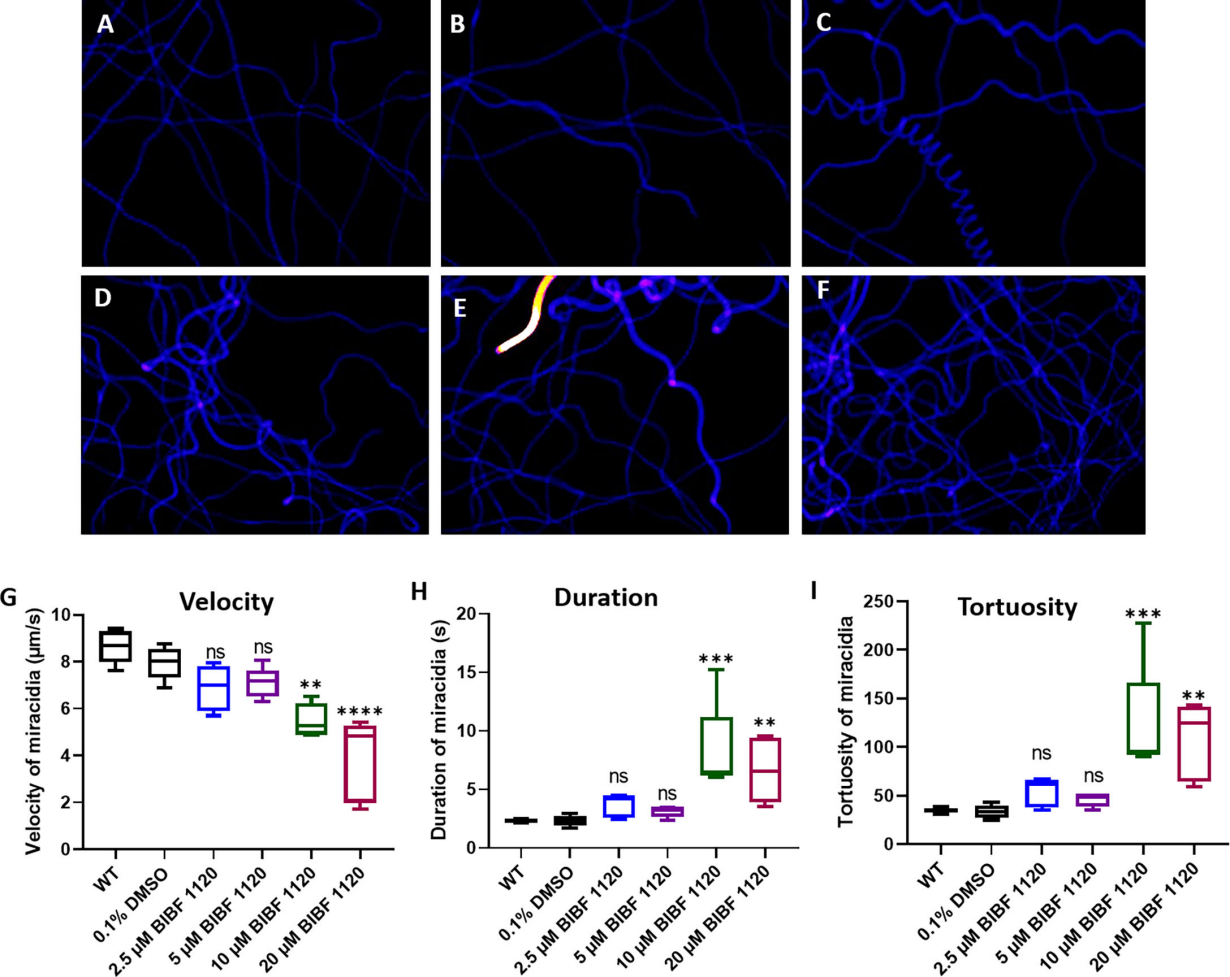
Behavioral changes in *S. mansoni* miracidia hatched from liver eggs treated with BIBF 1120. The behavior of miracidia hatched from WT eggs and eggs treated with 0.1% DMSO or BIBF 1120 at concentrations of 2.5 μM, 5 μM, 10 μM, and 20 μM was determined. Heatmaps **(A–F)** represent the movement patterns of individual miracidia within a 1 min recording. Colors in the heatmaps **(A–F)** show the time miracidia spent at a specific position. Black: absence; Blue: shorter time presence; Yellow and Red: longer time presence. Boxplots showing **(G)** velocity **(H)** duration, and **(I)** tortuosity of miracidial movement. (ns, not significant; **p value≤ 0.01; ***p value ≤ 0.001; ****p value ≤ 0.0001; One-way ANOVA).

Behavioral changes of miracidia hatched from eggs treated with BIBF 1120 were investigated by analyzing recordings of miracidial movement tracks. Heatmaps of the miracidia movement patterns were created to illustrate the behavior of individual miracidia within the 1 min recording. The heatmaps of the control miracidia [wild type miracidia (WT) and miracidia hatched from eggs treated with 0.1% DMSO] depicted linear soft blue lines suggesting these miracidia had less circular but faster movement (**Figures 9A, B**). In contrast, there were more circular lines and more abundant red and yellow regions in heatmaps of miracidia hatched from the BIBF 1120-treated eggs (**Figures 9C–F**), indicating more turning and circling and relatively slower movement of these miracidia in the FOV. As shown in **Figure 9G**, the swimming velocity of miracidia hatched from eggs treated with 10 μM and 20 μM BIBF 1120 was significantly decreased by 30.7% (*p*=0.0023) and 51.3% (*p ≤* 0.0001), respectively, compared with that measured in the 0.1% DMSO-treated group. Markedly, the average time duration of miracidia hatched from eggs treated with 10 μM and 20 μM BIBF 1120 in the FOV was elevated by 72.1% (*p*=0.0006) and 72.7% (*p*=0.0088), respectively (**Figure 9H**). Similarly, the movement tortuosity of miracidia hatched from 10 μM and 20 μM BIBF 1120 treated eggs was dramatically increased by 61.7% (*p*=0.0003) and 68.8% (*p*=0.0024), respectively, compared with that observed in the 0.1% DMSO-treated group (**Figure 9I**). No significant behavioral modifications were evident in miracidia hatched from eggs treated with BIBF 1120 at concentrations of 2.5 μM and 5 μM.

## Discussion

The somatic stem cell marker SmFGFRA has stimulated increased attention due to the essential roles it plays in maintaining schistosome stem cells (4–7, 36, 38). Transcription of *SmfgfrA* has been identified in the germinal cells (also defined as totipotent stem cells (5)) of miracidia, sporocysts and schistosomula (4, 6) and in the stem cells of adult *S. mansoni* (7, 39) [neoblasts or adult pluripotent stem cells (5)]. Recently, using single-cell RNA sequencing (scRNA-seq) analysis, Wendt *et al.* (39) characterized 68 distinct cell populations from adult *S. mansoni*, including three cell clusters expressing somatic stem cells markers (including *SmfgfrA*). They found the majority of *SmfgfrA* mRNA was detected in neoblasts and the tegument cells of adult schistosomes (39, 51). However, the precise functional roles of SmFGFRA in driving the development and differentiation of each developmental stage allowing them to thrive in diverse and challenging environments remains unclear. Herein, by immunolocalization and functional studies, we demonstrated abundant protein expression of SmFGFRA in various tissues of all examined *S. mansoni* life cycle stages and extended the range of prospective host-parasite cross-communication systems to the FGF family, reinforcing its likely multiple and critical functions in the survival and development of this flatworm parasite (6, 7, 27, 39).

In immature eggs, SmFGFRA was localized to all embryonic cells which possess large round nuclei. Specifically, the location was in the perinuclear region which is heavily involved in maintaining genome integrity and the regulation of gene expression (70), indicating this molecule is of critical importance in the differentiation and development of schistosome eggs. During development, the eggs develop extraembryonic envelopes beneath the shell and the embryonic cells differentiate into different schistosome tissues and organs (65). We showed that in mature eggs, the distribution of SmFGFRA extended to various tissues including the extra-embryonic von Lichtenberg’s layer, as well as the neural mass, lateral glands, the apical gland, and germinal cells of the miracidium. Of these, von Lichtenberg’s layer, which is present only in mature eggs, is highly active metabolically and is suggested to be the main source of immunogenic secretions that are released through eggshell pores into the host circulatory system to induce the host immune response (43, 71). This information supports the concept that SmFGFRA plays important roles in egg development and the host-parasite interplay.

The abundant expression of SmFGFRA in the neural mass of schistosome eggs and miracidia observed in our study and the transcription of *SmfgfrA* detected in almost all neuron cells of adult worms (39) suggest that expression of SmFGFRA in neurons occurs through all developmental stages of the parasite. SmFGFRA, as with its homologues in humans and mice (72–74), appears to promote neuronal cell development in schistosomes, but also plays a role in the continued activity of adult neurons. The inhibition of *S. mansoni* FGF signaling in schistosomes may affect neuronal development and function in schistosomes, and can provide the basis for developing an adjunct treatment to complement the currently widely used anti-schistosome drug, praziquantel, which induces severe but reversible contractions in worm muscles (75). Furthermore, clues to the potential importance of SmFGFA in neuronal function were evident by the remarkably changed behavior of miracidia hatched from BIBF 1120-treated eggs. The affected miracidia, which swim in freshwater by coordinated activity of cilia on their surface epithelial plates (76–78), displayed increased turning and circling motions in water. While the capacity of these affected miracidia to infect snails was not examined here, it seems improbable that they could navigate towards a *Biomphalaria* snail intermediate host in the natural aquatic environment after treatment with BIBF 1120 or a compound with comparable effects. Furthermore, treatment of *S. mansoni* eggs with BIBF 1120 resulted in a considerable reduction in egg hatching efficiency. These inhibitor-induced phenotype changes can be explained by the outcomes of a previous study showing the potential effects of BIBF1120 on mitotically active cells (such as proliferating cells or stem cells) in organs of adult *S. mansoni* (36), indicating the potential effect of BIBF 1120 in targeting SmFGFRA. BIBF 1120 is a selective inhibitor of mammalian FGFRs, vascular endothelial growth factor receptors (VEGFRs), platelet-derived growth factor receptors (PDGFRs) (79, 80), and Src-family kinases (81). Given the absence of VEGFR and PDGFR homologs in the genome of schistosomes, the SmFGFRs have been considered as the main targets of BIBF 1120 in these worms (36), a characteristic supported here by the capacity of BIBF 1120 to block the TK activity of SmFGFRA. However, a member of the Src family of cytoplasmic protein tyrosine kinases has been identified in *S. mansoni* (82) and further study is needed to investigate the effect of BIBF 1120 in regulating the functional activity of this and other Srcs in the blood fluke.

The presentation of SmFGFRA on the ciliated surface of newly released miracidia and internal cell masses (including the germinal cells), both of which are essential in the transformation of miracidia into primary sporocysts (6), implies a key role for SmFGFRA in promoting sporocyst growth and development. Miracidia penetrate the intermediate snail host resulting in the rapid shedding of ciliary epidermal plates from the larval surface during the development of the new tegumental syncytium of the developing sporocysts (83). Proteins (including SmFGFRA) in the epithelial cytoplasm, which are involved in the miracidium-to-sporocyst transformation (84), might also be important in modulating the immune response in the intermediate snail host, where cercariae are produced asexually and are released into fresh water as the next free-living stage of the life cycle (84). As expected, expression of SmFGFRA was detected in various tissues of cercariae including the tegument cytosol and oral sucker. Notably, a SmFGFRA-specific signal was also observed in the cercarial preacetabular glands which secrete enzymes for the penetration of mammalian host skin, in flame cells which are important for excretion processes (68, 85, 86), and in germinal cells. This pattern of localization suggests SmFGFRA is also associated with penetration of the mammalian host and in the transformation of cercariae to schistosomula. Importantly, the abundance of SmFGFRA in the germinal cells and tegument of cercariae would suggest that the molecule is produced in areas of rapid growth and differentiation in the development of schistosomula and further emphasize its critical role in the maturation of these juvenile worms into adults.

Given the pivotal role of stem cells in the renewal or repair of the tegument in adults (10, 87), the tegumental location of SmFGFRA in miracidia, cercariae, schistosomula and adult *S. mansoni* suggests the involvement of this protein in the process of stem cell - mediated tegumental regeneration. This is supported by scRNAseq data of cells isolated from adult worms showing *SmfgfrA* is transcribed in clusters of tegument 1 cells and tegument progenitor cells (39, 88). The tegumental location of SmFGFRA in adult schistosomes implies that SmFGFRA has potential as a vaccine and/or drug target, given the critical roles of tegumental proteins of schistosomes in the interplay with their mammalian hosts including hormone or nutrient uptake and immune evasion (89, 90). In addition, the pronounced expression of SmFGFRA in the testis of males and in the vitellaria of females, a stem cell-dependent tissue producing the yolk cells of eggs (39), suggests SmFGFRA is also heavily involved in egg production, reproductive system development and in the sexual maturation of adult schistosomes. These findings are also underpinned by the scRNAseq data which showed the presence of *SmfgfrA* transcripts in germline stem cells (GSC) and in vitellaria including vitellarium-specific stem cells (S1 cells) of both adult male and female worms, with higher levels in the S1 cells of females (39). To further determine the relationship between SmFGFRA and adult *S. mansoni* stem cells at the post-transcriptional level, we employed sequential labelling of EdU and using an anti-rSmFGFRA-L antibody in both males and female worms. We found all EdU^+^ stem cells expressed SmFGFRA, but not all cells expressing SmFGFRA were EdU^+^. This confirms the critical role of this protein in the functioning of schistosome stem cells and also highlights potentially multiple roles for SmFGFRA in *S. mansoni.*

To further explore the mammalian host and parasite interplay, we examined the conservation of FGF RTKs in *S. mansoni*, using 3D structure and domain structural analysis of SmFGFRA, SmFGFRB1 and SmFGFRB2. The similarity in structure between SmFGFRA and human mammalian FGFRs (27, 91) predicted a potential interaction between SmFGFRA and host FGFs. Since no *S. mansoni* endogenous FGF ligands have so far been identified, it is likely that activation of the SmFGFRA/FGF pathway in schistosomes is triggered by host FGFs, a scenario that is strongly supported by the protein interaction assay results we presented here showing SmFGFRA can bind the most active members of the human FGF family (26, 92, 93) - aFGF and bFGF, with much stronger binding affinity to the latter. Human bFGF is an essential exogenous growth factor required for maintaining the self-renewal of human embryonic stem cells (ESC) and induced pluripotent stem cells in culture (94–97). The strong binding affinity of human bFGF with SmFGFRA implies a crucial role for human bFGF in maintaining and promoting *S. mansoni* stem cell growth. We also demonstrated that the binding between human aFGF/bFGF and SmFGFRA could significantly induce the phosphorylation of Erk1/2 in adult worms of *S. mansoni*. The Erk signaling pathway regulates cell proliferation, differentiation and survival, and is one of the main MAPK pathways pivotal for sexual maturation of the female schistosome and egg production (98–100). The role of SmFGFRA in activation of the Erk pathway emphasizes again the importance of SmFGFRA in parasite development and maturation. Furthermore, given the plentiful presence of SmFGFRA in neurons of miracidia and germinal cells of miracidia and cercariae (two free-living stages), it remains to be determined whether there are other endogenous molecules that stimulate FGFR activity in these larval schistosomes.

Collectively, the results presented here emphasize the fundamental importance of SmFGFRA in driving the life cycle of *S. mansoni* and reveal its potential multiple functions in the proliferation of schistosome stem cells, in promoting cellular differentiation into tissues and organs, in the development of the nervous and reproductive systems, and in the host-parasite interplay. Our findings provide important clues for exploiting components of the FGF signaling pathway as promising targets for developing new interventions against schistosomiasis.

## Data Availability Statement

The original contributions presented in the study are included in the article/**Supplementary Material**. Further inquiries can be directed to the corresponding author.

## Ethics Statement

The animal study was reviewed and approved by Animal Ethics Committee (ethics number P242) of the QIMR Berghofer Medical Research Institute.

## Author Contributions

Conceived and designed the experiments: HY, XD and DM. Performed the experiments: XD and HY. Analyzed the data: XD, HY, CF, MJ, and DM. Contributed reagents/materials/analysis tools: XD and HY. Wrote the paper: XD, HY, DM and MJ. All authors contributed to the article and approved the submitted version.

## Funding

XD holds a Research Training Program (RTP) Scholarship and Graduate School Scholarship from the University of Queensland, Australia. DPM is a National Health and Medical Research Council (NHMRC) of Australia Leadership Fellow and receives Program (APP1132975), Project (APP1098244) and Investigator Grant (APP1194462) support from the NHMRC for his research on schistosomes and schistosomiasis. HY holds a QIMR Berghofer Medical Research Institute Seed Funding Grant (SF-210005).

## Conflict of Interest

The authors declare that the research was conducted in the absence of any commercial or financial relationships that could be construed as a potential conflict of interest.

## Publisher’s Note

All claims expressed in this article are solely those of the authors and do not necessarily represent those of their affiliated organizations, or those of the publisher, the editors and the reviewers. Any product that may be evaluated in this article, or claim that may be made by its manufacturer, is not guaranteed or endorsed by the publisher.
