# Supplementary material for: Schistosoma mansoni Fibroblast Growth Factor Receptor A Orchestrates Multiple Functions in Schistosome Biology and in the Host-Parasite Interplay
Source: Front Immunol. 2022 Jun 22;13:868077. doi: 10.3389/fimmu.2022.868077 (PMC9257043; doi:10.3389/fimmu.2022.868077)
Supplement: Supplementary file 1 [file DataSheet_1.pdf]

Supplementary Material

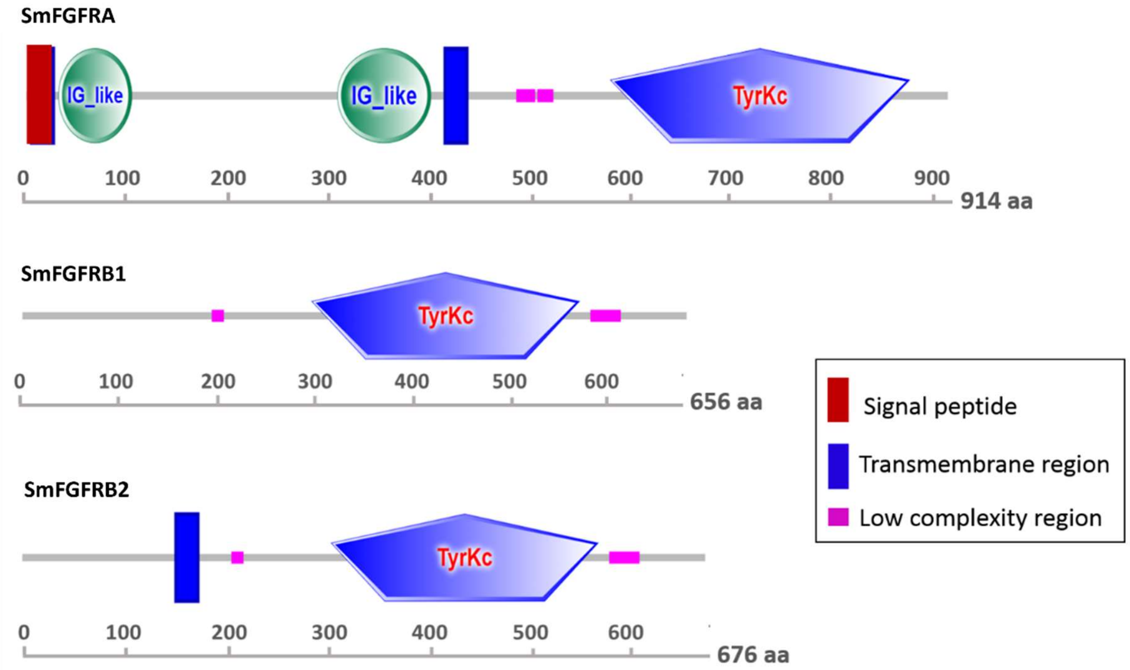

Supplementary Figure 1. Schematic representation of the domain structure of SmFGFRA, SmFGFRB1 and SmFGFRB2 analyzed by Simple Modular Architecture Research Tool (SMART). The positions and sizes of the predicted domains including an immunoglobulin (IG)-like domain (in green), tyrosine kinase (TK) domain (in purple), signal peptides (in red) and transmembrane domains (dark blue bars) are displayed. The low complexity region is shown in pink. The numbers of amino acids (aa) for each protein are shown at the bottom of each panel. SMART analysis showed SmFGFRA contains 914 aa, and is composed of a N-terminal signal peptide (M^{1} - G^{26}), two extracellular IG-like domains (positions Q^{32} - D^{105} and S^{310} - M^{403}) and an intracellular split TK domain (N^{582} - I^{880}) (Figure 1B). A TK domain was also identified in SmFGFRB1 (L^{286} - L^{550}) and SmFGFRB2 (L^{306} - L^{570}) while no signal peptide or IG-like domain were found in either SmFGFRB1 or SmFGFRB2.

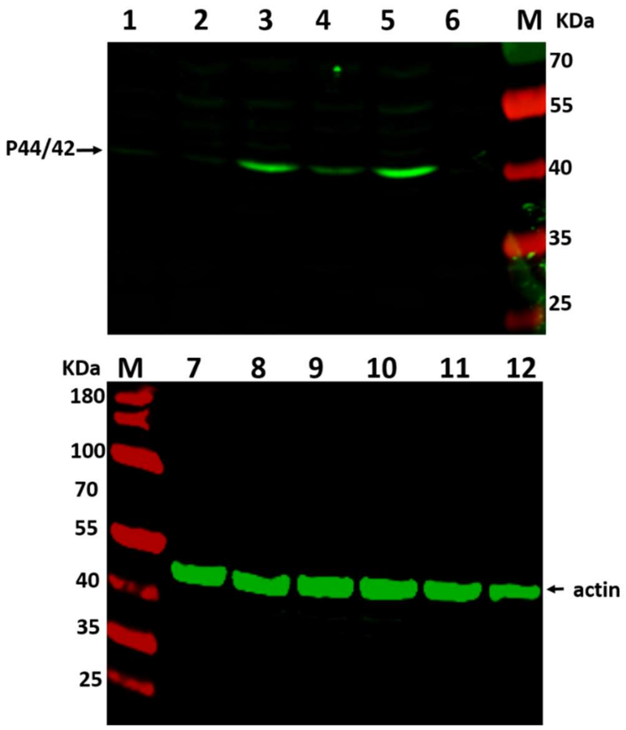

Supplementary Figure 2. Full image of **Figure 7 A.** Western blot analysis showing the effect of human aFGF or bFGF on stimulation of extracellular signal regulated kinases 1 and 2 (Erk1/2) in adult *S. mansoni* worms. An anti-phospho p44/42 MAPK (Erk) antibody was used to probe SWAP (soluble adult worm antigen preparation) of wild type (WT, untreated) adult worms (Lane 1) and worms which were incubated with 0.1% DMSO (Lane 2), human bFGF (Lane 3), human aFGF (Lane 4), both human bFGF and aFGF (Lane 5), and 10 µM BIBF 1120 (Lane 6). The intensity of actin expression in SWAP of WT worms (lane 7), and worms treated with 0.1% DMSO (Lane 8), human bFGF (Lane 9), human aFGF (Lane 10), both human bFGF and aFGF (Lane 11), and 10 µM BIBF 1120 (Lane 12) was evaluated, using an anti-actin antibody to ensure equal protein loading. The electrophoresed proteins used in the experiment were transferred for the western blot analysis to two PVDF membranes which were probed simultaneously with the anti-phospho-Erk1/2 antibody (upper panel) and the anti-actin antibody (bottom panel).
